# The effect of state gratitude on interpersonal trust under cognitive reappraisal among Chinese college students

**DOI:** 10.1186/s41155-024-00332-z

**Published:** 2024-11-21

**Authors:** Najam ul Hasan Abbasi, Li Zi Yi, Liu Yong, Mi Xue Xia, Abdul Hadi

**Affiliations:** 1 Department of Academic Sciences, Mianyang Normal University, Youxian District, Xianren Road No. 30, Section 1, Mianyang, Sichuan China; 2 Department of Psychology, University of Sindh, Jamshoro, Pakistan; 3 Department of Psychology, Guangxi University for Nationalities, Guangxi Zhuang Autonomous Region, Nanning, China; 4 Department of Sociology, Harran University, Sanliurfa, Turkey

**Keywords:** College students, Interpersonal trust, Cognitive reappraisal, State gratitude, Emotional regulation

## Abstract

**Objectives:**

This article starts with increasing emotions through cognitive reappraisal and explores the impact of state gratitude emotions on interpersonal trust among college students under cognitive reappraisal.

**Method:**

In study 1, we used a questionnaire survey and experimental design to collect data, which we processed/analyzed using SPSS 23.0. We employed, the Rotter Interpersonal Trust Scale (ITS), as adapted by Xu Huiyan, consisting of 25 items, to measure the interpersonal trust levels among college students and to explore the potentially significant differences in interpersonal trust levels among the students based on their demographic variables, including gender. We collected 347 survey questionnaires, with 143 from males and 204 from females, with a mean age of 20.93 (SD = 2.703). In study 2, we utilized a univariate inter-subject experimental design, with the independent variable being the state gratitude emotion (awakened vs. not awakened) under cognitive reappraisal, and the dependent variable was the level of interpersonal trust. Thirty participants participated in the pre-experiment, followed by 119 participants in the formal experiment to assess the impact of state gratitude emotion on interpersonal trust among college students, using motivation reappraisal strategy (*t* = − 4.028, *p* < 0.01).

**Results:**

This study found that (1) the overall interpersonal trust of college students was moderate to low (*M* = 38.01, SD = 9.13); (2) no significant differences in interpersonal trust levels were found among college students based on gender, grade, and whether they had children; however, the significant differences in interpersonal trust levels were observed among college students based on their the area of residence (*F* = 3.675, *p* < 0.05), school type (*F* = 3.04, *p* < 0.05), and family type (*t* = − 3.034, *p* < 0.01); (3) cognitive reappraisal has been shown to enhance individuals’ gratitude emotions in situations, thereby, raising their interpersonal trust level.

**Conclusions:**

We conclude that cognitive reappraisal enhances individuals’ positive emotions and levels of interpersonal trust in social interactions, offering a reference for promoting positive emotions and interpersonal trust levels among college students.

## Introduction

Interpersonal communication is the cornerstone of society’s normal operation, often involving interpersonal trust and relationships. Interpersonal trust, the trust formed between two people, is vital to the functioning of a social system. Trusting other people is important in negotiations (Denner et al., 2019; Kong et al., 2014; Olekalns & Smith, 2012; Savolainen & López-Fresno, 2018) and collaboration (De Dreu et al., 2006; Getha-Taylor et al., 2019). However, developing and maintaining interpersonal trust can be difficult (Williams & Belkin, 2016), especially in situations involving a stranger.

The college stage is an important period for the formation and development of interpersonal trust. Previous studies revealed a certain crisis of confidence in the communication process among college students’ interpersonal trust levels (He, 2022; Wang & Hu, 2021; 蔡力为, 2020). Recent studies in China on interpersonal trust among college students have mostly focused on analyzing the correlation between interpersonal trust and common variables, such as level of mental health and pro-social behavior, etc. (Guo, 2017; Yang et al., 2019). Although China’s traditional culture and modern education have always prioritized the development of integrity and interpersonal trust among people and students, various factors have negatively impacted interpersonal trust among college students ranging from the sociocultural transformation of Chinese society to the lack of self-awareness development t (He, 2022; 蔡力为, 2020). Trust is the lubricant of social life, and interpersonal trust runs throughout human society. Therefore, it is necessary to assess the interpersonal trust level among college students and improve their ability to adapt and respond to social changes.

Previous studies reveal that anger, happiness, sadness, gratitude, pride, and guilt can influence the initial level of trust among people/students (Angie et al., 2011; Dunn & Schweitzer, 2005). Researchers argue that gratitude, as a personality trait, is closely related to college students’ prosocial behavior and interpersonal trust (Angie et al., 2011; Grant & Gino, 2010). Most scholars divide gratitude into two types: trait gratitude and state gratitude. State gratitude is an effect that occurs after a person has been helped and that motivates the reciprocation of aid (Bartlett & Desteno, 2016; McCullough et al., 2001). From the perspective of emotional psychology, gratitude emotion is a kind of moral emotion, which refers to a kind of gratitude produced by individuals after receiving help. When gratitude becomes part of an individual’s personality, it is referred to as personal gratitude; in everyday situations, gratitude manifests in the form of emotional expression and is known as state gratitude. This emotional experience has the characteristics of closeness and often arises in specific situations, such as when receiving help from others. The focus of this research is on state gratitude and trust within interpersonal relationships, which makes the concept of gratitude more suitable for the expression of positive emotions. Thus, it entails an emotional experience including feelings of gratitude, joy, warmth, and desire for individuals following help from others, manifesting the immediate emotional experience of reward.

This study is based on the Positive emotion, Engagement, Relationships, Meaning, and Accomplishment (PERMA) theory within positive psychology, selecting state gratitude as the dependent variable. Proposed by Seligman (2011), this theory posits that positive emotions and strong interpersonal relationships are crucial to an individual’s sense of well-being. As a positive emotion, gratitude is closely associated with an individual’s happiness and life satisfaction (Bartlett & DeSteno, 2016; McCullough et al., 2001). Research indicates that feelings of gratitude can foster the development of positive personality traits, enhance the sense of social support, and elevate levels of interpersonal trust (Algoe & Haidt, 2009; Wood et al., 2010). For college students, cultivating a sense of gratitude significantly impacts their interpersonal trust levels. This demographic is at a pivotal transitional period in life, where social experiences and trust concepts can greatly shape their future life trajectories. Cultivating gratitude not only strengthens college students’ positive perceptions of others but also promotes prosocial behaviors in interpersonal interactions, thereby enhancing their interpersonal trust levels (Bartlett & DeSteno, 2016). Additionally, gratitude is correlated with the quality and durability of interpersonal relationships, facilitating the maintenance of long-term stable interpersonal connections (Algoe & Haidt, 2009). Research on the link between gratitude and interpersonal trust reveals that gratitude can promote the formation of interpersonal trust by enhancing individuals’ positive cognition and emotional experiences toward others (McCullough et al., 2001). These studies emphasize the importance of gratitude in building interpersonal trust and enhancing the quality of interpersonal communication among individuals. Thus, this study aims to assess how state gratitude impacts interpersonal trust levels among college students and how cultivating feelings of gratitude can promote their social interactions and psychological health.

Researchers have designed various forms of emotional regulation techniques that include cognitive reappraisal and expressive suppression. Although comparing these approaches has yielded crucial insights into the variations in emotional regulation mechanisms (Dillon et al., 2007; Goldin et al., 2008; Gross & James, 1998; Hayes et al., 2010; Sheppes & Meiran, 2007), it has under-emphasized the variability within each approach, such as that caused by varying goals (i.e., what people are trying to accomplish) or tactics (i.e., what people do). Cognitive reappraisal, a key emotional regulation strategy, involves adjusting emotional responses by altering the way we think. It is considered one of the most promising directions in the field of emotion regulation (Gross, 2015). Research indicates that cognitive reappraisal not only significantly affects individuals’ state gratitude emotions (Angie et al., 2011; Grant & Gino, 2010), but also positively impacts levels of interpersonal trust (Lount & Robert, 2010; Taylor & Heimberg, 2018).

This study proposes that cognitive reappraisal may mediate between state gratitude and interpersonal trust. By changing how individuals cognitively evaluate situations, cognitive reappraisal can not only enhance positive emotions such as gratitude but also potentially strengthen interpersonal trust. The cognitive reappraisal strategy posits that individuals can regulate their emotions by altering their cognitive evaluations of situations (or events) (Gross, 2015). Based on this theory, when individuals receive help, their positive evaluation of that help positively affects their state gratitude, which may, in turn, elevate their trust in others. Previous studies have found that cognitive reappraisal can change multiple aspects of emotional responses, including self-reported negative effects (Gross & James, 1998), peripheral physiological changes (Jackson et al., 2000; Ray et al., 2010), and neural indicators of emotional arousal (Hajcak & Nieuwenhuis, 2006; Ochsner et al., 2004; Urry et al., 2019). These findings provide a theoretical foundation for this study, supporting the notion that cognitive reappraisal is an effective emotion regulation tool that fosters the establishment of positive emotions and trust.

This study defines cognitive reappraisal as the process of changing emotions by altering the way we think about events. This strategy requires individuals to reinterpret and reassess situations that may elicit emotional responses, to form more positive emotional and cognitive experiences. This study seeks to investigate how cognitive reappraisal, as an emotion regulation strategy, can enhance state gratitude and subsequently raise interpersonal trust levels among college students, further validating its role in promoting individual psychological health and social adaptation. The focus on cognitive reappraisal as an intervention measure in this study is based on its theoretical and practical potential to impact gratitude and interpersonal trust.

Theoretically, cognitive reappraisal is one of the core strategies of emotion regulation, allowing individuals to regulate emotional responses by changing cognitive evaluations of situations (Gross, 2015). Empirical research has also shown that cognitive reappraisal can significantly enhance individuals’ positive emotional experiences, including gratitude (Grant & Gino, 2010), and is positively correlated with the development of interpersonal trust (Lount & Robert, 2010). Moreover, since cognitive reappraisal is a trainable/learnable skill, it serves as a feasible approach for intervention, offering potential applied value in promoting gratitude and interpersonal trust. This study will help fill the gap in the existing literature regarding empirical research on the impact of cognitive reappraisal on state gratitude emotions and interpersonal trust. Through experimental design, this study aims to provide direct evidence of the effects of cognitive reappraisal strategies on emotions and trust, thereby providing a scientific basis for applying cognitive reappraisal in promoting positive emotions and interpersonal trust. Through this research, we hope to offer new insights and methods for promoting individuals' social skills and psychological health, as well as provide a theoretical foundation and empirical support for future research.

## Methods

### Experimental research

#### Pre-experiment

To evaluate the feasibility of the experimental method, and identify necessary adjustments and modifications for the formal experiment, a pre-experiment was conducted. This pre-experiment intended to measure the effects of cognitive reappraisal on emotional states of state gratitude. The material was adapted from 张萍 and 张敏 (2016)’s state gratitude emotional awakening material to test whether the state gratitude material in the experiment can effectively activate the subject’s state gratitude emotion. Additionally, the study sought to assess differences in arousal emotion between participants receiving the cognitive reappraisal instruction and those given neutral instruction, thereby, exploring the influence of state gratitude on the change in interpersonal trust levels under cognitive reappraisal.

### Participants

A total of 32 undergraduates from a university in Sichuan, China, voluntarily participated in the experiment. This sample comprised 12 male and 20 female subjects. Two datasets were identified as invalid and subsequently excluded. After this exclusion, 30 valid datasets were retained for analysis, including 6 freshmen, 13 sophomores, and 11 juniors.

### Experimental materials

The experimental materials comprise typical stories encountered by college students where gratitude emotions may not be immediately obvious, and gratitude in these cases is mainly elicited through cognitive reappraisal. Initially, situational stories were articulated using neutral language to maintain objectivity and the length of the stories was standardized. After discussion with two psychology experts and psychology undergraduate students, their feedback was incorporated, thereby ultimately determining two situational stories.

1. Emotional priming materials for state gratitude: In this study, a paradigm of situational imagination was adopted for the priming of state gratitude. In this paradigm, subjects were given various situational stories and guided to immerse themselves in the situations. The situational imagination paradigm can only be effectively activated when the subjects are involved; therefore, two scoring questions were included for subjects to self-assess their level of immersion and emotional experience after reading each situational story. See the Appendix for the situational imagination materials and the scoring table for substitution.
2. Emotional check scale: Based on interviews with college students and discussions with experts, a subjective emotional experience evaluation scale was developed, reflecting the emotional themes of each situational story and indicators related to gratitude emotion experience indicators, namely the “sadness,” “complaint,” and gratitude adjective evaluation checklist (GAC).

GAC consists of three adjectives related to gratitude: “Grateful,” “Thankful,” and “Appreciative.” The evaluation employs a 5-point self-evaluation scale, where a higher O represents “completely absent” and 5 represents “particularly strong,” with higher scores indicating stronger emotional experiences. The score of this subjective emotional experience is divided into two parts: negative emotion score, which is the average score of various negative emotional experience intensity values reported by the subjects in a specific story), and gratitude emotion score, which is the average score of various gratitude emotional experience intensity values reported by the subjects in the same story).

Cognitive reassessment instruction for cognitive reappraisal uses the reinterpretation method (高丽华). Using specific prompts of the instructional language, the subjects’ state of gratitude was induced. Firstly, based on the operational definition of cognitive re-evaluation mentioned in the preface, two re-evaluation guidelines were created for the contextual content of the two stories. Secondly, through evaluations by psychological experts and interviews with students, a discussion was held on whether the instructional language reinterpreted the situation from a cognitive reappraisal perspective. Thirdly, a pre-experiment was conducted where college students reported whether their emotional experiences changed during the initial assessment and subsequent re-evaluation of the situational stories, and the effectiveness of the reevaluation guidance was repeatedly tested. Finally, based on the discussion results, modifications were made to the reevaluation guidance language, culminating in a finalized version. Once the instructions were determined, they were printed out for use in the formal experiment to avoid the subjective emotional impacts caused by personal/individual reading.

### Experimental process

The subjects were randomly divided into two groups: the state gratitude emotion arousal group and the neutral emotion group. In the pre-test stage, both groups read the same situational story and completed the situational substitution score sheet along with the emotion score checklist. In the stage of experimental operation, the state gratitude emotion arousal group read/received the cognitive reappraisal instruction; the neutral emotion group read/received the neutral instruction. In the post-test stage, both groups filled in the situational substitution scoring form and the emotional scoring checklist again. Finally, the subjects filled in the demographic information and guessed the purpose of the experiment. As a token of appreciation for their cooperation, a small gift was given to the subjects.

### Results and analysis

After excluding the questionnaires with missing answers, valid data was obtained from 30 subjects, and no one guessed the real purpose of the experiment. The valid questionnaires were coded and analyzed using the data processing software Statistical Package for Social Sciences (SPSS) 23.0, employing paired sample *t*-tests and other methods. The results showed that the 30 subjects demonstrated a high degree of substitution, indicating that the activation of the situational imagination paradigm was effective. By averaging the scores of the gratitude adjective rating checklist filled out by the subjects, it was found that the state gratitude induced by the cognitive reappraisal condition (*M* = 11.93, SD = 3.24) was significantly higher than that induced by the neutral material (*M* = 7.93, SD = 4.23, *t* (29) = − 2.741, *P* = 0.010 ≤ 0.01), which shows that the cognitive reappraisal method used in the experiment is effective in up-regulating state gratitude. Therefore, the pre-experimental results show that the manipulation of state gratitude is effective and can be used in formal experiments. See Tables 1, 2, and 3 below.

**Table 1:** Pre-experimental substitution execution degree score

	Cases	Minimum value	Maximum value	M	SD
Substitute the total score (pre-test)	30	3	16	10.267	3.3624
Substitute the total score (post-test)	30	2	18	10.767	5.0969

**Table 2:** Test of the priming effect of gratitude in the pre-experimental state

	Test group	Control group	t	*p*
GAC pre-test	7.60 ± 4.983	8.57 ± 3.877	− 0.583	0.565
GAC posttest	11.93 ± 3.240	7.93 ± 4.233	2.873	0.008
State gratitude D value	− 4.33 ± 2.820	0.64 ± 1.906	− 5.526	0.000

**Table 3:** Paired sample t-test before and after the awakening gratitude in the pre-experimental state

State gratitude (pretest)—state gratitude (posttest)	*t*	Degrees of freedom	Significance (two-tailed)
	− 2.741	29	0.010

### Formal experiment

#### Purpose of experiment

The influence of cognitive reappraisal on individual state gratitude is assessed to the level of interpersonal trust among individuals.

#### Experimental method

Using group testing and random sampling methods, we recruited college students from a certain university to voluntarily participate in the experiment. We contacted the participants to conduct the formal experiment in the classroom at a mutually agreed time. While maintaining a quiet environment, the participants carefully filled out the experimental questionnaire, which was then collected uniformly/collectively. After the experiment, each person was given a small gift. A total of 121 participants were selected for this experiment, but after eliminating invalid data, we obtained 119 valid questionnaires. The sample consisted of 40 male participants, and 79 female participants, with 32 freshmen, 20 sophomores, and 67 juniors, resulting in a mean of *M* = 20.7 and standard deviation of SD = 0.867. All participants had not previously participated in similar experiments, and they were informed of the purpose of this study only after the completion of the experiment. The experimental data was intended only for research purposes, and personal information would remain confidential.

#### Experimental materials

1. The experiment uses the trust game paradigm to create an investment game scenario (Powers & Labar, 2019). In this context, participants engaged in the task concurrently with another individual, adhering to the established rules of the trust game framework. The basic rules of the game are that the two parties involved are the investor and the investee. The investor has a certain amount of chips and can choose to invest part of the chips in the other party (investee). The chips received by the investor are equal to the amount given by the investor. Three times, the investor decides to return the amount to the investor, and the final investor’s income is the remaining chips and the investor’s returned chips. In the process of designing the experiment, the total amount of investment is set to 10 yuan, and the guideline is set as: part of the unknown players participating in the game at the same time is X yuan (0 yuan ≤ X ≤ 10 yuan), and the money sent will become Three times (3X yuan), the player who gets the money will return the investment to you within a certain period (the specific return amount is determined by the other party). For example, if you give the other party 5 yuan, the other party will get 15 yuan. At this time, the other party can return you Y yuan (0 yuan ≤ Y ≤ 15 yuan), and finally you will get the amount returned by the other player and your remaining sum of amounts. In this task, a larger amount of investment indicates a higher level of trust, and the amount of the investor is the dependent variable, which is the operational definition of interpersonal trust.
2. State gratitude emotion priming material. See the Appendix for the same pre-experimental materials.
3. Emotion detection scale, that is, the GAC scale and the imagined experience cognitive execution scale. See the Appendix for the same pre-experimental materials.

To make the research more scientifically rigorous and reliable, each participant is required to fill out an imagination experience task execution level checklist after the experiment. Question: To what extent do you immerse yourself in the protagonist of the scene during the reading process? How much emotion can you experience during the process of situational immersion? The execution level checklist adopts a 6-level rating system, ranging from 0 “completely absent” to 5 “very strong,” with higher values indicating greater levels of immersion and emotion. Participants who receive an average score below 3 will not be included in the experimental analysis.

(4) Cognitive re-evaluation instruction. See the Appendix for the same pre-experimental materials.

#### Experimental design

The purpose of the experiment is to investigate the effect of state gratitude on the interpersonal trust level of college students under cognitive reappraisal, using a single factor two-level between-subjects experimental design. The independent variable is the instruction to activate emotions, which is divided into two levels: cognitive reappraisal instruction and neutral instruction. The dependent variable is the final investment amount of the participants in the trust game task.

#### Experimental procedure

This experiment was conducted in a quiet classroom, using the double-blind method, control group, randomization, and other methods to control irrelevant variables. All recruited participants were randomly assigned to different groups and conducted in two different classrooms to ensure that participants were randomly assigned under experimental conditions. The experimental materials, investment game rules, and instructions are all presented in the form of paper questionnaires. The experiment is divided into three stages.

Before the experiment began, the main examiner explained to the subjects: “We are going to conduct a test. Everyone should keep quiet during the test, otherwise the research effect will be affected. The experiment will be conducted in another classroom at the same time. Please follow the instructions and complete the questionnaire step by step. You should not proceed to the second page until you have completed the first page.”

The first stage is the pre-test stage of the interpersonal trust level of college students. Participants read the rules of the investment game, and during this stage, they acted as investors and completed the game alongside another subject (false subject), that is, filling in the investment amount.

The second stage is the state gratitude emotion up-regulation manipulation stage.

- Task 1: Using the situational imagination paradigm, the subjects in the experimental group and the control group read the same situational story and completed the situational substitution score sheet as well as an emotion score checklist.
- Task 2: Use different instructions to manipulate the emotions of the subjects. Before the experiment started, the main experimenter informed the subjects to have a pen and white paper ready. To enhance the psychological involvement of the subjects and improve the effect of emotional priming, the subjects were asked to write down the content of the recalled situation on the paper.
- Task 3: The subjects read the situational story in the second stage of Task 1 again, and completed the situational substitution rating sheet and the emotional rating checklist.

The third stage is the post-test stage of the interpersonal trust level of college students. The subjects read the rules of the investment game again and refilled the investment amount after thinking about it.

Finally, the subjects filled in the demographic information and guessed the purpose of the experiment. As a token of appreciation for their active participation, each participant received a small gift (see Table 4).

**Table 4:**
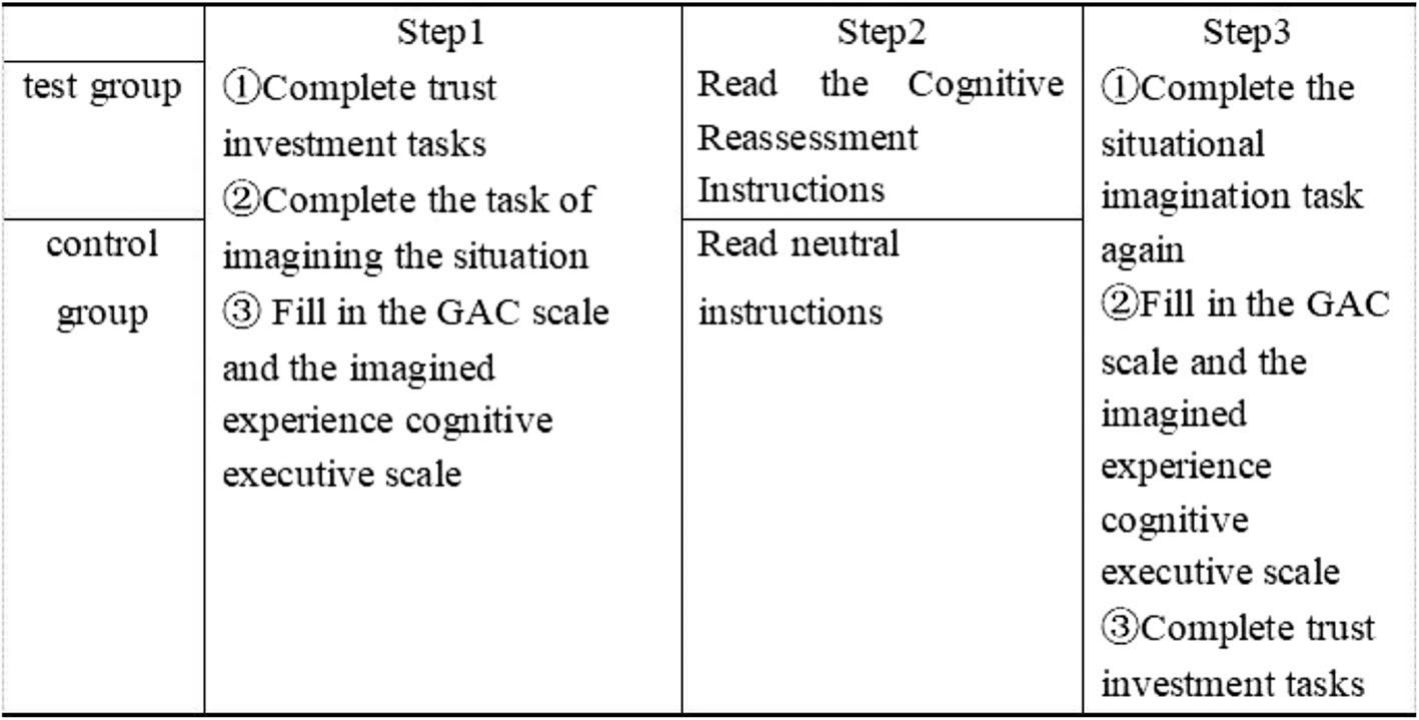
Flow chart of experimental design

## Results and analysis

Following the exclusion of invalid data, a total of 119 subjects provided valid responses and were included in the study; however, none of them discerned the true purpose of the experiment. The valid questionnaires were subsequently coded, and data analysis was performed. Among the sample, while 60 participants were placed in the cognitive reappraisal group, 59 participants were placed in the neutral group. The valid data were analyzed using SPSS version 23.0.

### (1) Validity test of status gratitude activation

An independent sample *t*-test and paired-sample *t*-test were used on the valid questionnaire data, showing that the up-regulation of state gratitude was effective. The results of state gratitude priming were evaluated using the Gratitude Adjectives Assessment Checklist (GAC), filled out by the subjects following the situational imagination task. Subjects in the experimental group showed a higher state gratitude emotion. The state gratitude induced by cognitive reappraisal instructions (*M* = 10.78, SD = 3.460) was significantly higher than that induced by neutral instructions (*M* = 6.53, SD = 3.923), *t* (117) = 6.282, *P* = 0.000 ≤ 0.01, indicating that after the subjects received the cognitive reappraisal instruction operation, their state gratitude was significantly up-regulated, and the up-regulation of the state gratitude was effective under the experimental conditions, see Tables 5 and 6.

**Table 5:** State gratitude priming effect test

	Test group	Control group	*t*	*p*
GAC pre-test	6.93 ± 3.813	7.08 ± 3.984	− 0.212	0.833
GAC posttest	10.78 ± 3.46	6.53 ± 3.923	6.282	0
State gratitude D value	− 3.85 ± 3.736	0.559 ± 2.010	− 7.997	0

**Table 6:** Paired sample t-test before and after emotional arousal of state gratitude

(Cognitive reassessment group) GAC pre-test—GAC post-test	*t*	Degrees of freedom	Significance (two-tailed)
	− 7.982	59	0.000

Analysis of the coded valid data using the independent sample *t*-test and paired-sample *t*-test showed that the main effect of state gratitude under cognitive reappraisal is significant, *t* (118) = − 2.933, *p* = 0.004 < 0.01. After the intervention of cognitive reappraisal, the investment amount of the participants in the gratitude-raising group (*M* = 6.30, SD = 2.374) was significantly higher than that of the participants in the neutral instruction group (*M* = 5.47, SD = 2.622). That is to say, the cognitive reappraisal instruction group has a significant improvement effect on the interpersonal trust level compared with the neutral instruction group. Therefore, increasing the state gratitude emotion can increase the investment amount of the subjects, and the state gratitude emotion under cognitive reappraisal has a significant effect on improving the interpersonal trust level of college students. In addition, there was no significant difference between the pretest trust scale (*M* = 5.46, SD = 2.555) in the neutral instruction group and the pretest trust scale (*M* = 5.32, SD = 2.175) in the cognitive reappraisal instruction group, *t* = − 0.324, *p* = 0.746 > 0.05, indicating that before receiving the experimental treatment, the two groups of subjects were homogeneous. The paired sample *t*-test was performed on the neutral instruction group, *t* (58) = − 0.077, *p* = 0.939 > 0.05, indicating that the neutral instruction in the experimental treatment did not change the emotions of the subjects without cognitive reappraisal operation level, the control operation is effective, see Tables 7 and 8.

**Table 7:** Test of difference in investment amount before and after cognitive reassessment

	Test group (M ± SD)	Control group (M ± SD)	*t*	*p*
Trust level pre-test	5.32 ± 2.175	5.46 ± 2.555	− 0.324	0.746
Trust Level Posttest	6.30 ± 2.374	5.47 ± 2.622	1.801	0.074
Trust level D value	− 0.98 ± 1.891	− 0.02 ± 1.697	-2.933	0.004

**Table 8:** Test of difference in investment amount after cognitive reappraisal intervention

	M ± SD	*t*	*p*
Cognitive reappraisal team	− 0.983 ± 1.891	− 4.028	0
Neutral steering group	− 0.017 ± 1.697	− 0.077	0.939

## Discussion

The main finding of this study is that the cognitive reappraisal strategy can effectively enhance state gratitude emotions and subsequently improve the level of interpersonal trust among college students. The results are consistent with Gross’s emotion regulation theory, which posits that individuals can regulate emotional responses through cognitive reappraisal. Our study found that the cognitive reappraisal strategy can effectively increase state gratitude emotions, thereby enhancing the level of interpersonal trust among college students. This not only verifies the applicability of Gross’s theory in the field of interpersonal trust but also extends the empirical research of this theory in the field of positive emotions and social behavior. The results of our study are consistent with existing theoretical frameworks, that is, positive emotions (such as gratitude) can promote individuals’ social behavior and trust tendencies. Through experimental design, we found that state gratitude emotions under the cognitive reappraisal strategy can significantly improve individuals’ levels of interpersonal trust, which is consistent with previous studies on the positive impact of positive emotions on interpersonal trust. The experimental results verified the experimental hypotheses and confirmed the Gross Emotion Regulation Theory, which posits that individuals can reinterpret events that cause emotions and change emotional responses through cognitive reappraisal strategies (Taylor & Heimberg, 2018). Zheng and Hu’s (2019) research on the psychological structure of trust, including cognitive evaluation, was also confirmed in this experiment, indicating that cognitive evaluation affects interpersonal trust and also confirms that intervention studies can effectively enhance interpersonal trust. Yuan Bo and others proposed that compared with neutral emotions, positive emotions can promote interpersonal trust to a certain extent, which was also verified in this experiment. Gruber et al. (2014) conducted a cognitive reappraisal experiment on bipolar affective disorder subjects and found that cognitive reappraisal involvement had a significant effect on regulating positive emotions, that is to say, when individuals are involved in related situations, adopting a positive cognitive reappraisal strategy can not only improve the level of positive emotions but also improve the level of interpersonal trust. This finding indicates that specific psychological intervention methods can actively influence individuals' emotional states and social behaviors, highlighting their important practical significance for promoting positive emotions and establishing trusting interpersonal relationships. Cognitive reappraisal provides a mechanism to regulate emotional responses by changing individual cognitive evaluations of situations, which is beneficial for individual mental health and social adaptation. The ability to increase state gratitude emotions and, consequently enhance interpersonal trust levels through cognitive reappraisal strategies provides new insights into the dynamics and plasticity of the relationship between gratitude emotions and interpersonal trust.

## Conclusion

From the analysis results of the formal experiment, it can be seen that after the subjects received the cognitive reappraisal instructions, the subjects’ state of gratitude improved significantly, and the amount of investment in the investee increased. The third stage of the experiment was the last investment of the subjects. It reflects the degree of trust the subject has in the other party, indicating that compared with the subjects in the neutral emotion control group, state gratitude under cognitive reappraisal can improve the interpersonal trust level of college students.

The results of study 2 confirmed the experimental hypothesis, demonstrating that cognitive reappraisal can enhance individual state gratitude in a situation. Additionally, the effect of state gratitude under cognitive reappraisal was found to be more significant than under neutral conditions in improving interpersonal trust. In various situations, individuals can change their emotions through cognitive reappraisal, using positive and grateful emotions to improve the level of interpersonal trust and promote the development of personal relationships.

### Advantages and limitations

In this study, we explored the connection between state gratitude emotions and interpersonal trust, based on the PERMA theory, which posits that the cultivation of positive emotions is an effective way to enhance individual well-being. Employing a cognitive reappraisal strategy, this study successfully elevated state gratitude emotions by altering individual cognitive evaluations of situations. The upregulation of this emotion not only intensified participants’ positive emotional experiences but also significantly increased their sense of interpersonal trust. This was reflected in the research findings: when individuals experienced higher levels of state gratitude, they were more inclined to trust others and more willing to establish and maintain positive interpersonal relationships. Additionally, the results revealed the central role of relationship orientation in Chinese interpersonal interactions. Relationship orientation refers to the emphasis individuals place on establishing and maintaining relationships in social interactions (Liang Hongyu et.al, 2015), echoing the social function of positive emotions in the PERMA theory. As Bartlett and DeSteno pointed out, relationship-based trust is more likely to promote individuals' prosocial behavior (Hu Yu & Sun Dengyong, 2010). The findings of this study provide empirical support for this theoretical perspective, indicating that by enhancing state gratitude emotions, not only can individuals/ positive emotional experiences be intensified, but also their expression of prosocial behavior can be fostered.

The sample of this study is primarily composed of college students, which aids in understanding the mechanisms of interpersonal trust and emotion regulation within this specific group, but it also limits the generalizability and external validity of the findings. College students are generally at a similar stage of development, and their cognitive and emotional regulation capabilities may differ from those of adults in other stages. Consequently, the results of this study may not reflect the levels of interpersonal trust among people of different ages from various cultural and educational backgrounds. Future research could include participants from different age groups, such as adolescents and middle to older adults, to explore the manifestations of interpersonal trust and emotion regulation across various life cycles. Conducting studies across different cultural contexts can assess how cultural differences impact the relationship between interpersonal trust and emotion regulation. Additionally, diversifying the sample populations by including individuals with varying educational levels and socioeconomic statuses can enhance the universality and transferability of the research findings.

The study utilized the trust game paradigm, enabling researchers to observe and quantify interpersonal trust behavior within a controlled environment, providing a relatively authentic and intuitive method for assessing the level of interpersonal trust. Through cognitive reappraisal manipulation, the study can directly observe the impact of emotion regulation strategies on interpersonal trust, offering an empirical basis for understanding the role of emotion regulation in the formation of interpersonal trust. The experimental design mitigates the influence of confounding variables through random assignment and controlled conditions, increasing the internal validity of the research findings. However, this method relies on participants’ self-reporting, which may introduce biases in the assessment of emotional states and interpersonal trust, such as social desirability effects. The experimental results may be partially explained by other unmeasured emotional variables; participants may experience other positive emotions, like hope or optimism, which can also affect interpersonal trust. In future research, different experimental conditions can be set, such as simulating various social environments, to evaluate the impact of environmental factors on interpersonal trust. Additionally, potential confounding variables can be controlled by incorporating additional measurements, such as assessing participants’ pre-existing trust tendencies and emotional states.

### Suggestions for future research

1. Expand the research group of subjects, such as teenagers who are in the critical period of forming interpersonal attitudes, and middle-aged and elderly people who have rich life experience, to obtain more real, systematic, and scientific research results, and explore the relationship between different age groups and groups. Different influencing factors on the level of trust can make more targeted suggestions while enriching the research results.
2. The findings of this study highlight the potential of cognitive reappraisal techniques in promoting positive emotions and interpersonal trust. Future research can leverage these discoveries to design and implement effective intervention measures. By teaching individuals how to use cognitive reappraisal to improve communication, educational institutions can incorporate cognitive reappraisal techniques into courses and counseling programs to enhance students' social skills and sense of trust. In therapeutic contexts, cognitive reappraisal can be part of psychotherapy to assist patients in managing emotions and establishing healthier interpersonal relationships. Applying cognitive reappraisal in conflict situations can help individuals view issues from a more positive perspective, reducing misunderstandings and conflicts. Utilizing cognitive reappraisal to assist individuals in better managing emotions and mitigating the impact of negative emotions can strengthen interpersonal relationships and improve individual well-being.
3. Future research can explore various emotion regulation strategies, such as expressive suppression and problem-focused coping, and how they affect interpersonal trust. Studies across different age groups and cultural backgrounds can reveal how these variables change over time and differ across cultures. The application of physiological measurements and neuroscience methods can provide biomarkers for the connection between emotion regulation and interpersonal trust. Long-term follow-up and intervention studies can assess the long-term impact of emotion regulation on interpersonal trust. The use of virtual reality technology can offer new practical tools for emotion regulation. Multidisciplinary approaches can foster a deeper understanding of the complex relationship between emotion regulation and interpersonal trust. These research directions can not only advance theoretical development but also provide guidance for practical applications, enhancing individual and societal well-being.

## Data Availability

The datasets processed and analyzed during the current study are available from the corresponding/first author on reasonable request.

## Appendix 1. Survey on interpersonal trust among college students

**Table:**

Question content	totally agree	Partially agree	Neutral	Partially disagree	absolutely disagree
1. Hypocrisy is becoming more and more common in our society	1	2	3	4	5
2. You'd better be careful when dealing with strangers unless they provide evidence that they are trustworthy	1	2	3	4	5
3. Not having the teacher present to supervise the exam may lead to more people cheating	1	2	3	4	5
4. The United Nations will never become an effective force in maintaining world peace	1	2	3	4	5
5. Courts are places where we can all be treated in public	1	2	3	4	5
6. No matter what people say (state), it is best to think that most people are primarily concerned with their own happiness	1	2	3	4	5
7. Although news can be seen in newspapers, radio, and television, it is difficult to get objective reports on public events	1	2	3	4	5
8. The future seems promising	1	2	3	4	5
9. Most elected officials are sincere in their campaign promises	1	2	3	4	5
10. Most people will definitely implement their plans if they tell them	1	2	3	4	5
11. In this competitive era, if you are not vigilant, others may take advantage of you	1	2	3	4	5
12. Most idealists are sincere and act in accordance with the creed they profess	1	2	3	4	5
13. Most salespeople are honest when describing their products	1	2	3	4	5
14. Most students will not cheat even when they are sure they will not be caught	1	2	3	4	5
15. Most maintenance personnel will not charge more even if they think you do not understand their professional knowledge	1	2	3	4	5

## Appendix 2. Trust Game Task

This is an investment and trading game. You currently have 10 yuan in your hand. If you are willing to give another stranger player who participates in the game at the same time a part of the money X yuan (0 yuan ≤ X ≤ 10 yuan), the money given away will become three times (3X yuan), and the player who got the money will return the investment to you within a certain period of time (the specific return amount is determined by the other party). For example, if you give the other player 5 yuan, the other party will get 15 yuan. At this time, the other party can return you Y yuan (0 yuan ≤ Y ≤ 15 yuan). In the end, you will get the amount returned by the other player and what is left by yourself. The sum of the amounts.

Now, please think about it, how much of the 10 yuan are you willing to give to the other party?

Please write down the amount you are willing to give the other party: Yuan.

## Appendix 3. Scenario Stories and Cognitive Reassessment Instructions

Situational Story 1: Today is my first day at the university. Carrying large and small bags alone, I finally completed the admission procedures. I walked into the dormitory exhausted and saw the parents of my roommates helping them make their beds and luggage.

Test group.

Scenario 1: Imagine that your parents accompany you to school and help you prepare your bed and luggage. When you encounter more problems and difficulties in the future, you always hope to have your parents by your side, relying on your parents and having their help. At the beginning of school, your parents let you complete the admission procedures and sorting out your studies on your own, hoping to cultivate your independent character, which will also help you face more difficulties alone in college life. Now please try to read the following situation from this perspective.

Control group.

Now please carefully recall the layout of the school, such as where the teacher’s office building is located, how many entrances the school has, the directions of each entrance, how many teaching buildings there are, what sports equipment or entertainment facilities are on the playground, daily life Where to go through the school, etc. Please quietly recall and feel, and simply write down what you recall on a white paper.

## Appendix 4. Cognitive Evaluation Scale

Please rate the degree to which you took on the role and experienced emotions in Situations 1 and 2 respectively. A score of 0 means no feeling at all, a score of 5 means a very strong feeling. The larger the value, the stronger the degree of involvement or experience. Please rate the corresponding value. Put a “√” on it.

1、 During the reading process, to what extent did you become the protagonist of the situation?

Scenario 1: Your parents did not send you to college 0 1 2 3 4 5.

Scenario 2: The traffic police punish you for violating the rules 0 1 2 3 4 5.

2、 In the process of situational substitution, to what extent can you experience the emotions at that time?

Scenario 1: Your parents did not send you to college 0 1 2 3 4 5.

Scenario 2: The traffic police punish you for violating the rules 0 1 2 3 4 5.

## Abbreviations

PERMA: Positive emotion, engagement, relationships, meaning, and accomplishment
GAC: Gratitude adjective evaluation checklist
SPSS: Statistical package for social sciences
